# Parvovirus B19-associated erythropoiesis-stimulating agent hyporesponsiveness in a patient on maintenance hemodialysis: A case report

**DOI:** 10.3892/mi.2026.337

**Published:** 2026-08-24

**Authors:** Theodoros Eleftheriadis, Maria Divani, Maria-Anna Polyzou-Konsta, Evangelos Lykotsetas, Christina Poulianiti, Andriani Balatsouka, Ioannis Stefanidis

**Affiliations:** Department of Nephrology, Faculty of Medicine, University of Thessaly, 41110 Larissa, Greece

**Keywords:** Parvovirus B19, hemodialysis, anemia, erythropoiesis-stimulating agent hyporesponsiveness, intravenous immunoglobulin, iron deficiency

## Abstract

Parvovirus B19 is an uncommon cause of hypoproliferative anemia. In maintenance hemodialysis (HD), anemia is rarely explained by a single mechanism, and the viral suppression of erythropoiesis may be overlooked when infection, inflammation and iron deficiency occur concomitantly. The present case report describes the case of a 79-year-old man receiving chronic HD who was admitted twice with prolonged fever and was eventually treated for culture-negative mitral valve endocarditis. During the second hospitalization period, the hemoglobin level decreased from 8.8 to 7.7 g/dl, despite an increase in the level of recombinant human erythropoietin (rHuEPO) from 30,000 to 60,000 IU/week. The number of reticulocytes decreased from 2.07 to 0.95%, and a blood transfusion was required. The patient tested positive for Parvovirus B19 immunoglobulin M and immunoglobulin G, and polymerase chain reaction (PCR) confirmed active infection. Bleeding, hemolysis, vitamin B12 and folate deficiency, uncontrolled hypothyroidism, malignancy and inadequate dialysis were excluded. Intravenous immunoglobulin was administered at 0.4 g/kg/day for 5 days. By the time of discharge, the hemoglobin level had increased to 9.4 g/dl and reticulocytes had increased to 2.7%. Repeat PCR at follow-up, 20 days following discharge, yielded negative results. Severe iron deficiency was then evident, and following the administration of ferric carboxymaltose, the hemoglobin level further increased to 12.1 g/dl, allowing a reduction in rHuEPO. On the whole, the present case report underlines that Parvovirus B19, although rare, should remain in the differential diagnosis of erythropoiesis-stimulating agent-resistant anemia in patients undergoing HD.

## Introduction

Anemia is common in patients with end-stage kidney disease (ESKD) who receive maintenance hemodialysis (HD). Although reduced endogenous erythropoietin production is the central mechanism, the observed anemia is usually multifactorial. Absolute or functional iron deficiency, inflammation, blood loss, shortened red-cell survival, nutritional deficiencies and marrow hyporesponsiveness may all contribute to its development (1,2). The majority of patients, therefore, require erythropoiesis-stimulating agent (ESA) therapy, often in conjunction with iron supplementation. When the hemoglobin level decreases despite adequate or increased ESA treatment, the differential diagnosis should remain broad and include iron deficiency, inflammation or infection, occult bleeding, hemolysis, vitamin B12 or folate deficiency, hypothyroidism, inadequate dialysis, hyperparathyroidism, malignancy, marrow disease, anti-erythropoietin antibody-mediated pure red cell aplasia (PRCA), and infections that directly suppress erythropoiesis (1,2).

Human parvovirus B19 has a strong tropism for erythroid progenitor cells and may abruptly interrupt red-cell production (3). In immunocompetent individuals, infection is usually self-limited. However, in patients with limited erythropoietic reserve, it may cause a clinically significant transient aplastic crisis. In immunocompromised patients, persistent infection can lead to chronic PRCA (3). Overt parvovirus B19-associated erythroid suppression has rarely been reported in patients undergoing dialysis (4-6).

The present case report describes the case of a patient on maintenance HD with prolonged fever, culture-negative mitral-valve endocarditis and parvovirus B19 infection confirmed by serology and polymerase chain reaction (PCR). The patient developed a disproportionate, ESA-resistant hypoproliferative anemia, followed by severe iron deficiency during hematological recovery.

## Case report

A 79-year-old man on maintenance HD was admitted to the University Hospital of Larissa (Larissa, Greece) for the evaluation of recurrent fever. His medical history included ESKD, right nephrectomy for urothelial carcinoma 8 years prior, a bladder tumor treated with transurethral resection and intravesical mitomycin C 5 years prior, arterial hypertension, abdominal aortic aneurysm repair and hypothyroidism. Preceding the episode described herein, he was receiving recombinant human erythropoietin (rHuEPO) at 10,000 IU three times weekly (30,000 IU/week).

The first hospitalization period followed ~1 week of fever. Blood and urine cultures had already been obtained in the HD unit, and empirical antibiotics (ceftazidime IV, 1 g after each HD session) had been commenced for suspected urinary infection due to a recent episode of urinary retention and catheterization. During hospitalization, blood and urine cultures remained sterile. Transthoracic echocardiography revealed no vegetation, and chest imaging did not reveal an infectious focus. He received meropenem (1 g/day) and teicoplanin (400 mg after each HD session) and gradually became afebrile. Upon admission, the hemoglobin level was 10.1 g/dl, and the C-reactive protein (CRP) level was 0.27 mg/dl. At discharge on day 6, the hemoglobin level was 9.3 g/dl and the CRP level was 0.29 mg/dl. The rHuEPO dose remained at 30,000 IU/week, and he was discharged afebrile with instructions to continue intravenous antimicrobial treatment during HD.

Fever recurred soon after discharge despite ongoing antimicrobial therapy, and amikacin (initially at 3 mg/kg IV after each HD session, targeting trough levels of 5 mg/l) was added prior to readmission. At the second hospitalization, new blood and urine cultures were obtained, and empirical treatment was changed to daptomycin (500 mg IV after each HD session), piperacillin/tazobactam (2.25 g IV every 8 h) and metronidazole (500 mg IV every 8 h); doxycycline (100 mg/day PO) was added after 5 days. This antibiotic treatment continued until the end of treatment for endocarditis, i.e., for 8 weeks. Repeated blood cultures remained negative, most likely as the patient had been continuously exposed to antibiotics before and during the admission. Testing for *Clostridioides difficile* yielded negative results and urine culture was sterile, *Legionella* urinary antigen was negative, and the assessment of the arteriovenous fistula did not suggest access infection. Immunological testing did not support systemic autoimmune disease.

**Figure 1:**
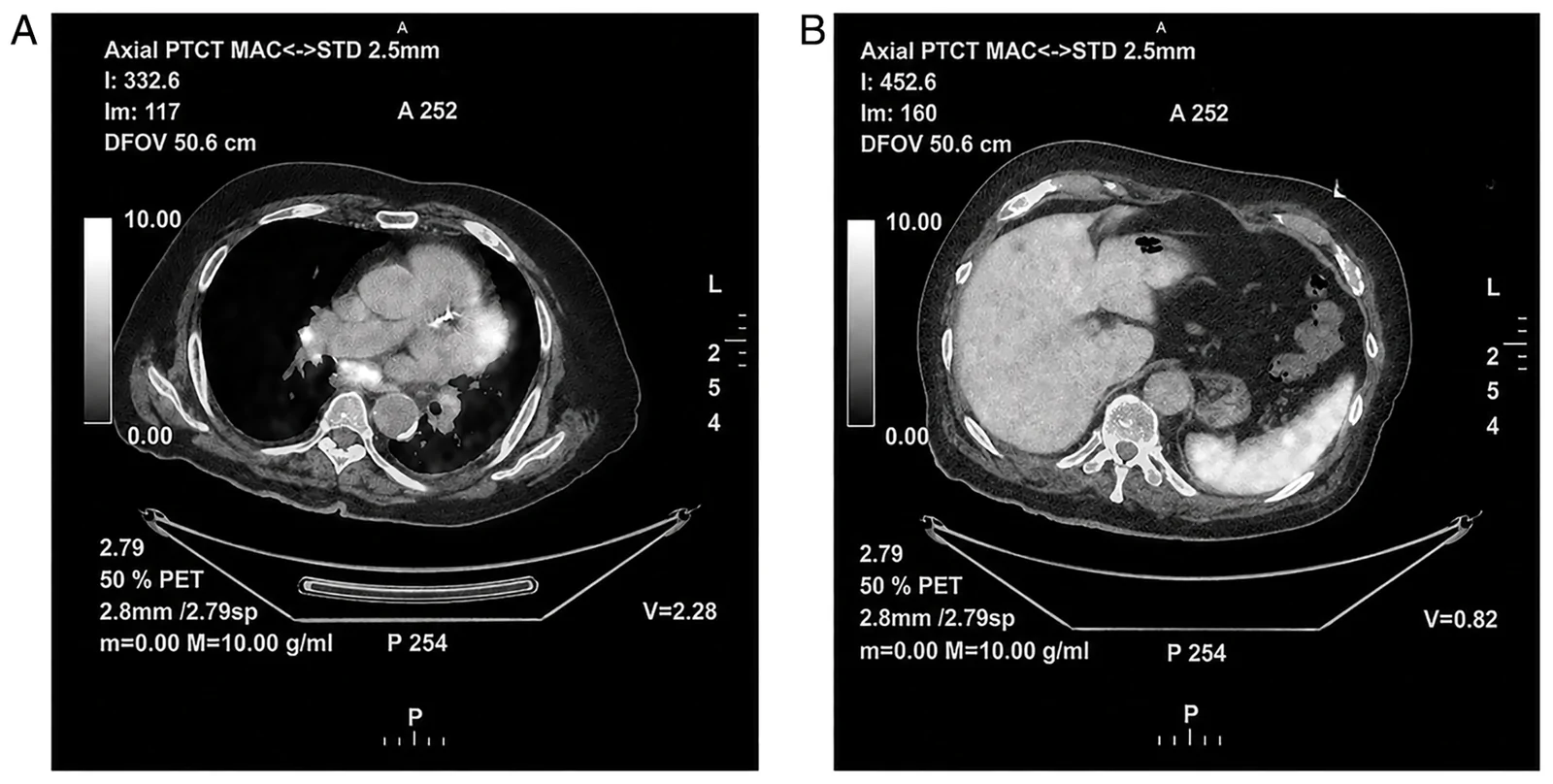
18F-FDG PET/CT images. (A) Axial chest image demonstrating hypermetabolic hilar/mediastinal lymph nodes associated with diffuse infiltrative-type pulmonary 18F-FDG uptake. (B) Axial upper abdominal image demonstrating diffusely increased splenic 18F-FDG uptake. 18F-FDG, axial fused fluorodeoxyglucose F 18.

As the fever persisted, transesophageal echocardiography was performed, which revealed findings consistent with mitral valve endocarditis. The patient was therefore managed as having culture-negative endocarditis. Positron emission tomography/computed tomography (PET/CT) was performed due to the history of urothelial malignancy, persistent fever and concern for occult infectious, inflammatory, malignant, or aortic-graft involvement. PET/CT revealed hypermetabolic lymph nodes, diffuse pulmonary infiltrative-type uptake and splenic uptake (Fig. 1). These findings were interpreted as inflammatory or infectious in origin. Thoracic and lumbar spine magnetic resonance imaging, requested for low back pain and tenderness, did not reveal any evidence of spondylodiscitis.

During the second period of hospitalization, the fever resolved and inflammatory activity decreased; however, anemia continued to worsen despite ESA therapy. On day 1, the hemoglobin level was 8.8 g/dl, the CRP level was 3.46 mg/dl, and rHuEPO was 30,000 IU/week. On day 6, the hemoglobin level was 8.2 g/dl, reticulocytes were 2.07%, the reticulocyte index was 1.13, the reticulocyte production index was 0.57, the CRP level was 3.17 mg/dl, the ferritin level was 207 ng/ml, and transferrin saturation (TSAT) was 13.14%. Due to the ongoing decrease in the hemoglobin level, rHuEPO was increased to 20,000 IU three times weekly (60,000 IU/week). On day 19, the hemoglobin level was 7.7 g/dl, reticulocytes had decreased to 0.95%, the reticulocyte index was 0.49, the reticulocyte production index was 0.24, the CRP level was 0.99 mg/dl and transfusion was required. Iron was not administered due to active infection.

The decrease in reticulocytes from 2.07% on day 6 to 0.95% at the hemoglobin nadir was considered inappropriately low for the degree of anemia and the level of ESA exposure, prompting testing for Parvovirus B19. Parvovirus B19 serology was positive, with elevated immunoglobulin M (IgM) antibodies (28.58 U/ml; positive cut-off, >15 U/ml) and immunoglobulin G (IgG) antibodies (8.9 IU/ml; positive cut-off, >5 IU/ml), as measured using enzyme-linked immunosorbent assay (ELISA) with the SERION ELISA classic Parvovirus B19 IgM and IgG assays (Institut Virion/Serion GmbH; cat. nos. ESR122M and ESR122G, respectively). Subsequently, parvovirus B19 deoxyribonucleic acid (DNA) was detected in plasma by the qualitative interpretation of a real-time PCR assay using the commercially available kit, Parvovirus B19 Real-TM Quant (Sacace Biotechnologies, Como, Italy; Cat. No. V49-50FRT), confirming active infection.

Other common causes of ESA hyporesponsiveness were evaluated. There was no active bleeding or positive fecal occult blood test. Hemolysis was not supported as the direct antiglobulin test was negative, and haptoglobin was normal. Vitamin B12 and folic acid levels were normal. The hypothyroidism of the patient was under control with levothyroxine, since the level of serum thyroid-stimulating hormone (TSH) was within the laboratory reference range (0.27-4.20 mIU/l). The negative PET-CT along with normal serum alkaline phosphatase (ALP) and lactate dehydrogenase (LDH) levels and the absence of leukoerythroblastic features on peripheral blood smear examination rendered malignant cell bone marrow infiltration unlikely. Dialysis adequacy was acceptable, with a urea reduction ratio of ~68%. The low TSAT suggested iron-restricted erythropoiesis as a possible contributor, but intravenous iron was not given during active infection and endocarditis treatment.

Intravenous immunoglobulin (IVIG) was administered at 0.4 g/kg/day for 5 days. At the time of discharge, on day 30 of the second period of hospitalization, the hemoglobin level was 9.4 g/dl, reticulocytes had increased to 2.7%, the reticulocyte index was 1.69, the reticulocyte production index was 1.13, the CRP level had decreased to 0.15 mg/dl and rHuEPO was continued at 60,000 IU/week. He remained afebrile and was discharged with instructions to complete antimicrobial therapy for culture-negative endocarditis.

During the post-discharge antibiotic period, rHuEPO was maintained at 60,000 IU/week, and intravenous iron was deferred due to recent endocarditis and ongoing antibiotic therapy. At follow-up at 20 days following discharge, the hemoglobin level was 10.5 g/dl, reticulocytes were 1.8%, the reticulocyte index was 1.26, the reticulocyte production index was 0.84, the CRP level was 0.4 mg/dl, the ferritin level was 32.9 ng/ml and TSAT was 9.07%, indicating a marked iron deficiency. Repeat parvovirus B19 PCR was negative.

**Figure 2:**
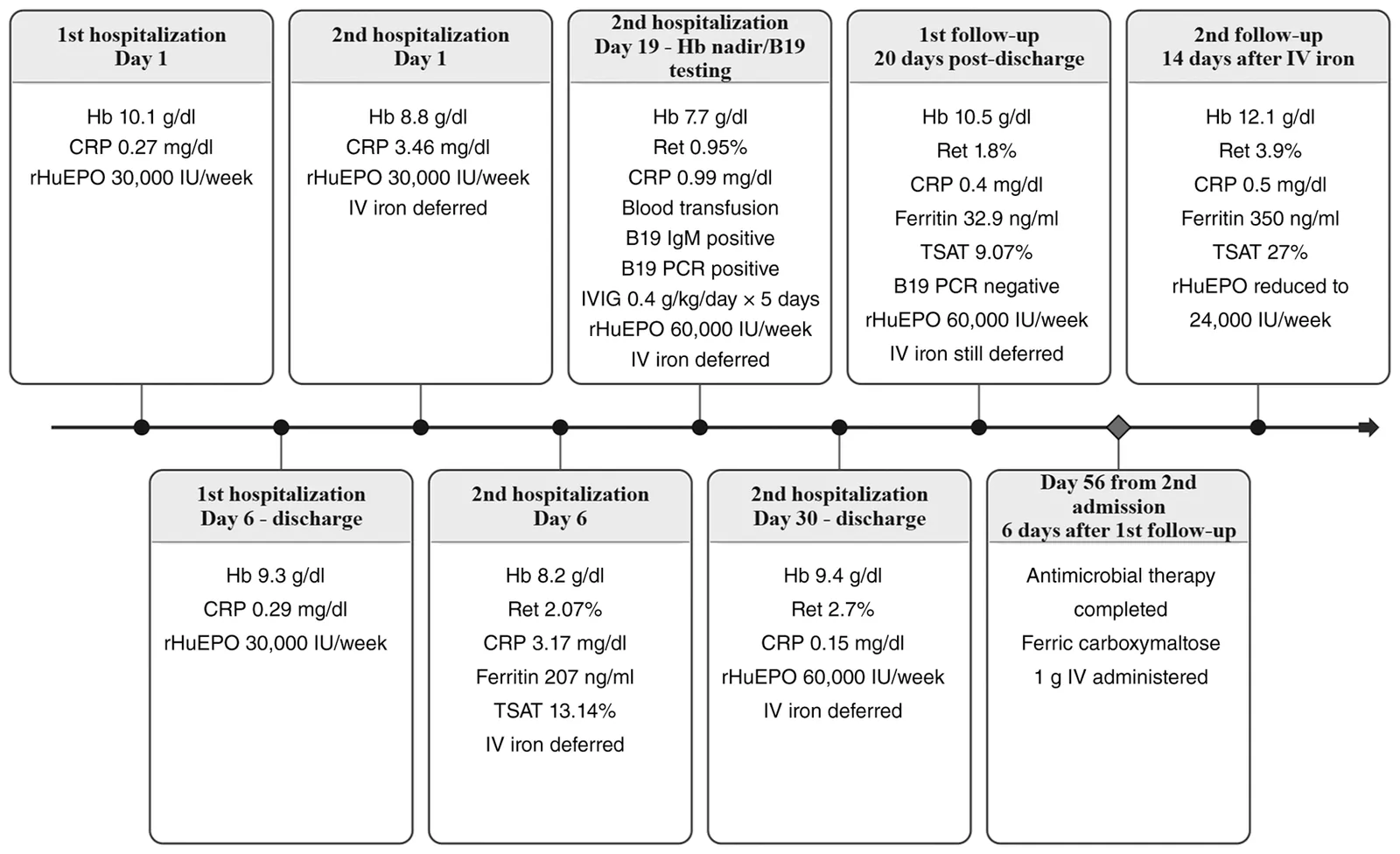
Simplified clinical course of Parvovirus B19-associated anemia in a patient on maintenance hemodialysis. Boxes summarize Hb (g/dl), Ret (%), CRP (mg/dl), ferritin (ng/ml), TSAT (%), Parvovirus B19 serology and PCR results, rHuEPO dose, IVIG administration, blood transfusion and follow-up findings. Hb, hemoglobin; Ret, reticulocytes; CRP, C-reactive protein; TSAT, transferrin saturation; PCR, polymerase chain reaction; rHuEPO, recombinant human erythropoietin; IVIG, intravenous immunoglobulin.`

At 6 days after this follow-up, on day 56 of the second hospitalization period, the patient completed an 8-week course of antimicrobial therapy. On the same day, he received 1 g of intravenous ferric carboxymaltose. At 14 days thereafter, the ferritin level had increased to 350 ng/ml, TSAT increased to 27%, the hemoglobin level increased to 12.1 g/dl, reticulocytes increased to 3.9%, the reticulocyte index increased to 3.15, the reticulocyte production index increased to 3.15, and the CRP was 0.5 mg/dl. The rHuEPO dose was then reduced to 8,000 IU three times weekly (24,000 IU/week). The simplified clinical course of the patient is presented in Fig. 2.

## Discussion

The present case report describes a diagnostically difficult episode of ESA-resistant anemia in a patient undergoing HD. The patient had a prolonged fever and was treated for culture-negative mitral valve endocarditis. In this setting, a decrease in the hemoglobin level can easily be attributed to infection, inflammation and reduced iron availability. In this patient, however, the sequence of events suggested that inflammation and iron restriction were not the only explanations.

The hemoglobin level decreased despite the doubling of the rHuEPO dose from 30,000 to 60,000 IU/week. The reticulocyte response was inadequate, decreasing from 2.07% on day 6 to 0.95% at the hemoglobin nadir. Parvovirus B19 infection was supported by IgM and IgG serology and confirmed by PCR. Other major non-iron causes were not identified. There was no overt or occult bleeding, the direct antiglobulin test was negative, haptoglobin was normal, vitamin B12 and folic acid levels were normal, TSH was within the laboratory reference range, and dialysis adequacy was acceptable. In addition, PET-CT did not detect malignancy. Although PET-CT cannot completely rule out bone marrow infiltration, it has good diagnostic discriminatory ability in this setting (7,8). Serum ALP and LDH were within the normal range, and the peripheral blood smear revealed no leukoerythroblastic features, further supporting the absence of marrow infiltration by malignant cells. Following IVIG, reticulocytes increased to 2.7%, and repeat PCR 20 days after discharge yielded negative results.

The observed decrease in reticulocytes is consistent with the known erythroid tropism of Parvovirus B19. The virus infects erythroid progenitor cells and can cause transient aplastic crisis (3,9). Persistent infection may lead to chronic PRCA in immunocompromised patients (3,10). Reports on patients undergoing non-transplant HD are limited. Duranay *et al* (4) described a patient undergoing HD with fever, arthralgia, severe anemia, very low reticulocytes, positive Parvovirus B19 IgM, and worsening anemia despite an increased erythropoietin dose. Ozeki *et al* (5) reported a patient on HD with rapidly progressive anemia and severe reticulocytopenia, with positive Parvovirus B19 IgM and PCR. Shi *et al* (6) described an adult patient on peritoneal dialysis with chronic PRCA, erythroid hypoplasia in the marrow, positive IgM, high viral load and improvement following IVIG. A heart-transplant recipient on chronic HD has also been reported, although transplant-related immunosuppression makes that case clinically distinct (10).

The present case report resembled previous dialysis reports in several respects, including ESA dependence, worsening anemia, inadequate reticulocytosis, exclusion of bleeding and hemolysis and subsequent hematologic recovery. There were also differences from the earlier dialysis cases. The patient described herein was elderly, and his ESKD state was complicated by endocarditis. Adaptive immunity is impaired in patients undergoing HD (11). Although the patient was not receiving immunosuppressive medications, the addition of a severe infection to his advanced age and HD state is likely to further suppress his immune system, rendering him vulnerable to Parvovirus B19 infection. In addition, he had an iron deficiency. Iron was not administered due to the infection, and following IVIG treatment, the iron deficiency became severe, likely due to increased iron utilization during hematologic recovery. As expected, hemoglobin levels and ESA responsiveness improved only partially following IVIG, whereas a more complete hematologic response was observed only following iron administration and the completion of antibiotic treatment for endocarditis.

As regards Parvovirus B19 serology, IgM typically appears in the 2nd week following infection and may persist for several months, while IgG appears shortly thereafter and usually persists lifelong (12,13). In immunocompetent individuals, false-negative IgM results are uncommon, although false-positive IgM results from commercial assays have been reported (14). Therefore, when available, PCR is useful for a definitive diagnosis. This is particularly critical in immunocompromised patients, such as solid-organ transplant recipients (15), in whom false-negative IgM results can reach 25% (16). In the present case reprot, positive PCR supported the serological diagnosis, and subsequent PCR negativity supported virological resolution.

The main difficulty in interpreting the case described herein was the coexistence of multiple causes of anemia. During hospitalization, the decline in reticulocytes, a positive parvovirus B19 PCR, the need for transfusion, and an early reticulocyte rise following IVIG all support a diagnosis of acute viral erythroid suppression. The patient was iron-deficient, and after discharge, the deficiency became severe, possibly due to its utilization for erythropoiesis following treatment for Parvovirus B19. The ferritin level was 32.9 ng/ml and TSAT was 9.07% while the patient was still receiving high-dose rHuEPO. Intravenous iron was deferred during the active systemic infection, in accordance with current guidelines for anemia management in chronic kidney disease (17). It was administered on day 56 after the start of the second hospitalization, once the infection had clinically resolved and the eight-week antimicrobial course was completed. At 14 days following the administration of ferric carboxymaltose, iron indices improved, hemoglobin increased to 12.1 g/dl, reticulocytes increased to 3.9%, and the rHuEPO dose could be reduced. Taken together, the clinical course suggests that several factors were contributing at the same time. Parvovirus B19 appears to have played a role in the acute suppression of erythropoiesis, while iron deficiency became a key contributor to the persistent ESA hyporesponsiveness during recovery.

In addition to iron deficiency, infection likely contributes to the observed anemia. In a patient with endocarditis, the initial decrease in hemoglobin could reasonably be explained by inflammation, restricted iron availability and reduced responsiveness to ESA therapy. It is also recognized that the suppressive effects of inflammation on erythropoiesis may continue even after fever has resolved and CRP has begun to decline (18). However, as the condition of the patient evolved, inflammation alone no longer appeared to explain the progressive hypoproliferative anemia. Between days 6 and 19 of the second hospitalization, the patient became afebrile, and the CRP level decreased from 3.17 to 0.99 mg/dl. During the same period, the hemoglobin level continued to decrease from 8.2 to 7.7 g/dl, while the reticulocyte percentage declined from 2.07 to 0.95% and the reticulocyte production index from 0.57 to 0.24. This worsening of erythropoiesis occurred despite doubling the rHuEPO dose from 30,000 to 60,000 IU/week, prompting the investigation of other possible causes. Positive parvovirus B19 IgM serology, together with detectable viral DNA in plasma, indicated a recent active infection. Following the administration of IVIG, the reticulocyte percentage increased to 2.7% and the reticulocyte production index to 1.13. Parvovirus B19 PCR later yielded negative results. This sequence does not prove a direct causal association. However, the worsening reticulocytopenia in the presence of parvovirus B19 DNAemia, followed by reticulocyte recovery following IVIG and subsequent PCR negativity, suggests that parvovirus B19 contributed to the suppression of erythropoiesis. The anemia was therefore most likely multifactorial. Inflammation related to endocarditis and impaired iron availability contributed to ESA hyporesponsiveness, while Parvovirus B19 appears to have contributed to an additional acute suppression of red-cell production. The further improvement in hemoglobin following intravenous iron administration also highlights the critical role of iron deficiency during the recovery period.

IVIG has been used for parvovirus B19-related erythroid suppression, particularly when viral clearance is impaired (19). In the patient described herein, reticulocytes recovered following IVIG and follow-up PCR became negative, whereas a more complete hemoglobin response occurred only after iron deficiency was corrected. Clinically, this sequence is critical. IVIG was followed by reticulocyte recovery and PCR negativity, although the correction of iron deficiency was still required for a fuller hemoglobin response.

The present case report has certain limitations. One such limitation is that neutralizing anti-EPO antibodies were not measured; thus, antibody-mediated PRCA cannot be completely ruled out. However, the clinical course of the patient rendered this diagnosis less likely. In suspected ESA-related PRCA, the Kidney Disease: Improving Global Outcomes (KDIGO) Anemia Work Group recommends discontinuing ESA therapy, providing transfusion support when needed, and considering immunosuppressive treatment. IVIG is not specifically recommended (17). Similarly, in a retrospective study of 47 patients with epoetin-induced PRCA, only 11% of those treated with IVIG achieved hematological recovery, while recovery was considerably more common with immunosuppressive therapy (20). Although improvement following IVIG does not exclude antibody-mediated PRCA, the overall pattern in the patient described herein was more suggestive of parvovirus B19-associated erythroid suppression. Parvovirus B19 DNA was detected, the reticulocyte percentage increased from 0.95 to 2.7% after the 5-day course of IVIG, and repeat PCR subsequently became negative. Moreover, rHuEPO treatment was continued, which would prevent hematological recovery in epoetin-induced PRCA. The dose was eventually reduced after correction of iron deficiency. The authors therefore considered neutralizing anti-EPO antibody-mediated PRCA less likely. Another limitation is that bone marrow examination was not performed. Quantitative parvovirus B19 viral-load measurements were also not available. Therefore, although the combination of PCR positivity, reticulocytopenia, subsequent reticulocyte recovery and PCR negativity supports a clinically relevant role for Parvovirus B19, its contribution cannot be separated with certainty from that of inflammation and iron restriction. Parvovirus B19 infection should be considered in patients undergoing HD with an unexpected decrease in hemoglobin levels and ESA hyporesponsiveness, particularly when the reticulocyte response is inappropriately low, or the severity of anemia seems disproportionate to the inflammatory state. Rather than relying on a specific reticulocyte or hemoglobin threshold, testing may be particularly useful when progressive anemia and marked reticulocytopenia persist despite increasing ESA exposure and appear disproportionate to the course of inflammation, after more common causes of ESA hyporesponsiveness have been evaluated. The impaired immunity associated with HD may increase vulnerability to Parvovirus B19 infection, while the multifactorial nature of anemia in this population can delay diagnosis. Early recognition is critical as IVIG may be followed by the recovery of erythropoiesis.

## Data Availability

The data generated in the present study may be requested from the corresponding author.
